# Positive Regulation of Splicing of Cellular and Viral mRNA by Intragenic RNA Elements That Activate the Stress Kinase PKR, an Antiviral Mechanism

**DOI:** 10.3390/genes14050974

**Published:** 2023-04-26

**Authors:** Raymond Kaempfer

**Affiliations:** Department of Biochemistry and Molecular Biology, Institute of Medical Research Israel-Canada, The Hebrew University-Hadassah Medical School, Jerusalem 9112102, Israel; kaempfer@hebrew.edu

**Keywords:** mRNA splicing, intragenic RNA elements, intracellular stress response, stress kinase PKR, TNF, viral mRNA splicing, HIV

## Abstract

The transient activation of the cellular stress kinase, protein kinase RNA-activated (PKR), by double-helical RNA, especially by viral double-stranded RNA generated during replication, results in the inhibition of translation via the phosphorylation of eukaryotic initiation factor 2 α-chain (eIF2α). Exceptionally, short intragenic elements within primary transcripts of the human tumor necrosis factor (*TNF-α*) and *globin* genes, genes essential for survival, can form RNA structures that strongly activate PKR and thereby render the splicing of their mRNAs highly efficient. These intragenic RNA activators of PKR promote early spliceosome assembly and splicing by inducing phosphorylation of nuclear eIF2α, without impairing the translation of the mature spliced mRNA. Unexpectedly, excision of the large human immunodeficiency virus (HIV) *rev/tat* intron was shown to require activation of PKR by the viral RNA and eIF2α phosphorylation. The splicing of *rev/tat* mRNA is abrogated by viral antagonists of PKR and by *trans*-dominant negative mutant PKR, yet enhanced by the overexpression of PKR. The *TNFα* and HIV RNA activators of PKR fold into compact pseudoknots that are highly conserved within the phylogeny, supporting their essential role in the upregulation of splicing. HIV provides the first example of a virus co-opting a major cellular antiviral mechanism, the activation of PKR by its RNA, to promote splicing.

## 1. Introduction

Intracellular stress induces phosphorylation of the α-chain of eukaryotic translation initiation factor 2 (eIF2α) [1,2]. The phosphorylation of eIF2α on Ser51 blocks the GDP/GTP exchange that is critical for allowing the eIF2 molecule, once it has been released from the mRNA after an initiation event, to return to the state that enables it to start a new round of initiation of translation [3]. A prominent eIF2α kinase is protein kinase RNA-activated (PKR). PKR is a serine/threonine protein kinase that depends on RNA for its activation. Without RNA-mediated activation, this kinase is expressed in the cell in latent, non-active form. Activation of PKR is a critical aspect of the antiviral state that is induced in the cell upon exposure to the interferons (IFNs). Double-stranded RNA activates PKR, which enables the kinase to phosphorylate eIF2α; eIF2α phosphorylation then inhibits the initiation of mRNA translation, resulting in apoptosis of the infected cells that acts to limit virus spread [4,5]. To enable the *trans*-autophosphorylation that is necessary for activation of the kinase, two molecules of PKR must transiently associate on the activating RNA molecule, thereby forming a homodimer [6,7]. This implies that PKR must bind to the activating RNA with moderate affinity, so that it can be released promptly once activation has taken place. Indeed, the binding of PKR to RNA with high affinity can prevent the activation of the kinase, which emphasizes the importance of a transient interaction. For example, by binding tightly to PKR, adenovirus VA RNA sequesters PKR in an inactive state and thereby is able to inhibit activation of the kinase [8].

Typically, PKR is activated upon virus infection by double-stranded RNA that is generated during virus replication. Recently, however, it was revealed that viral double-helical RNA (either from replication of the viral RNA genome or from symmetric transcription of the viral DNA genome) is not the exclusive inducer of PKR activation. Cellular genes can harbor short RNA sequences that are able to control gene expression at mRNA translation or at splicing of the encoded mRNA through the activation of PKR and the induction of eIF2α phosphorylation [9,10]. Thus, a sequence within human *interferon-γ (IFN-γ)* mRNA that consists of its 5′-untranslated region (5′-UTR) and the first 26 codons can fold into an RNA pseudoknot that, exceptionally, is capable of activating PKR within the cytoplasm, eliciting the phosphorylation of eIF2α [9,11]. This enables *IFN-γ* mRNA to downregulate its own translation, which prevents the excessive synthesis of this inflammatory cytokine that otherwise could cause pathology [9,11]. During translation in the cell, the *IFN-γ* mRNA structure undergoes dynamic refolding that enables it to function both as activator of PKR and as translation template [11].

The normally accepted function of PKR, once activated, is to inhibit protein synthesis. However, the splicing of tumor necrosis factor-α (*TNF-α*) mRNA precursor transcript (pre-mRNA) was demonstrated to depend on the ability of this pre-mRNA to activate PKR [10,12,13], a *cis*-acting RNA element that is located within the 3′-UTR of the *TNF-α* pre-mRNA folds into a pseudoknot that can strongly activate PKR [10,13]. Activation of the kinase renders the splicing of *TNF-α* mRNA highly efficient [10,13]. The splicing of *TNF-α* mRNA is fully dependent on the phosphorylation of eIF2α; however, that dependence does not cause translational repression of the spliced mRNA [10]. The regulation of mRNA splicing through the activation of PKR and phosphorylation of eIF2α is not limited to the inflammatory response. The splicing of human *α-globin* and *β-globin* pre-mRNA depends strictly on the activation of PKR by intragenic RNA elements, as well as on the phosphorylation of eIF2α within the cell nucleus [14]. The *TNF-α* and *globin* genes are the only cellular genes for which this mechanism of splicing control has been demonstrated thus far. Indeed, it is exceptional: although closely related to *TNF-α*, the *TNF-β* gene does not harbor an RNA sequence that is able to activate PKR, reflecting the majority of genes [10,13]. As a result, the splicing of *TNF-β* mRNA occurs with an efficiency that is an order of magnitude lower than that of *TNF-α* mRNA [10].

The activation of PKR in the cell and phosphorylation of eIF2α are transient events. The *Trans*-autophosphorylation of PKR needed for kinase activation and phosphorylation of eIF2α are both local, transitory events in the cell that occur in close proximity to the activating RNA molecule, and are followed promptly by dephosphorylation [15,16,17]. Once dephosphorylation has taken place, PKR returns to its inactive state while eIF2α becomes active again and can fulfill its role in the initiation of protein synthesis. The overall amounts of activated PKR and of phosphorylated eIF2α within the cell do not change. This accounts for the *cis*-acting nature of the intragenic *TNF-α* RNA element [10]. When the *TNF-α* RNA element was inserted into the *TNF-β* 3’-UTR, that was sufficient to increase the splicing efficiency of *TNF-β* mRNA by an order of magnitude [10]. Thus, an RNA element within the gene capable of activating PKR can locally render splicing far more efficient. The localized activation of PKR accounts also for the *cis*-acting nature of the *IFN-γ* RNA activator of PKR, which does not cause a global inhibition of translation in the cell, but is selective for the synthesis of IFN-γ [9].

The activation of PKR by the viral double-stranded RNA generated during virus replication and the phosphorylation of eIF2α yields the inhibition of the translation of the viral mRNA that is considered to be a key aspect of the antiviral response of the cell [4,5]. However, recent work has revealed that, surprisingly, a virus can use this prominent cellular antiviral mechanism to its advantage to achieve efficient mRNA splicing. In an outstanding example, both the activation of PKR and the phosphorylation of eIF2α are indispensable for enabling the splicing of human immunodeficiency virus-1 (HIV) *rev/tat* mRNA [18]. Thus, HIV adopted this cellular antiviral mechanism for its own benefit.

Here, we review the molecular mechanisms underlying PKR-dependent mRNA splicing for the first examples of this gene control: *TNF-α*, *α-globin* and *β-globin*, and HIV *rev/tat*. An earlier review [19] covered aspects of the PKR-dependent splicing of *TNF-α* and *globin* mRNA that created the basis for the discovery that the splicing of HIV *rev/tat* mRNA also requires the activation of PKR and phosphorylation of eIF2α. Notably, in each case, the mechanism differs in detail. For example, whereas a single RNA element activates PKR in the case of the *TNF-α* and *globin* genes, there are two PKR-activator elements within HIV RNA, one of which is dominant. The RNA elements that activate PKR show commonality as well as distinction in both structure and function. The comparative analysis in this review aims to achieve greater insight into an entirely novel mechanism of RNA-mediated gene regulation.

## 2. Positive Regulation of Splicing of *TNF-α* mRNA by an RNA Element within the Pre-mRNA That Activates PKR, Inducing Nuclear eIF2α Phosphorylation

**Figure 1:**
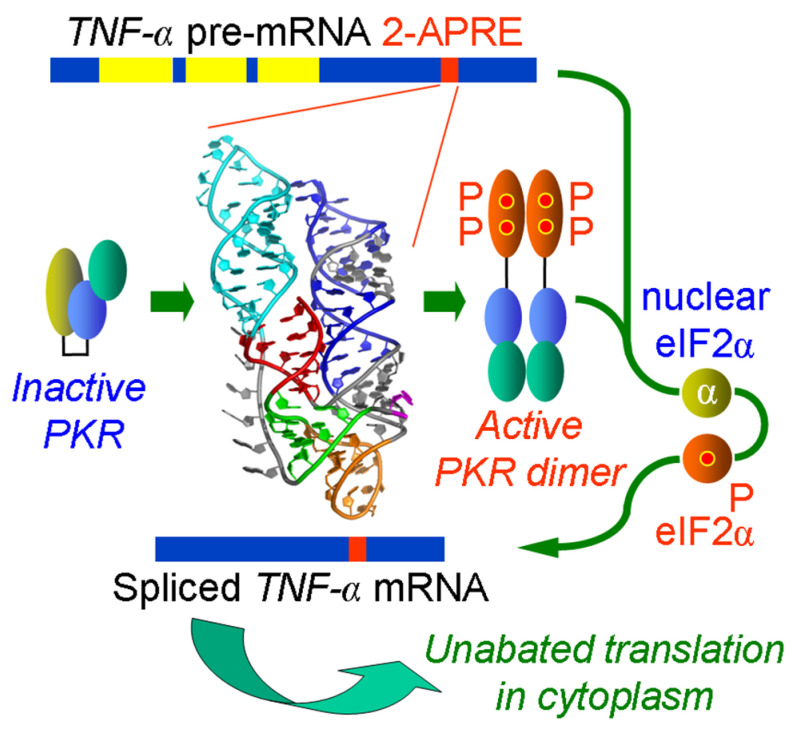
Regulation of splicing of *TNF-α* pre-mRNA by an intragenic element that induces activation of PKR, resulting in eIF2α phosphorylation. Exons are in dark blue. The PKR activator element of 104 nucleotides in length (2-APRE) that maps into the *TNF-α* 3′-UTR (red) strongly activates PKR within the nucleus [13]. This RNA element folds into a pseudoknot structure that causes the RNA to be constrained into two parallel helices, each capable of binding to a single PKR monomer, thereby facilitating the formation of PKR dimer on the 2-APRE RNA that is needed for the activation of the kinase [10]. The active PKR dimer next must induce the phosphorylation of nuclear eIF2α in order to enable the highly efficient excision of the introns (yellow) from *TNF-α* pre-mRNA [10]. The mature *TNF-α* mRNA splicing product is exported from the nucleus into the cytoplasm and is translated without impediment [10,13,19].

The inflammatory cytokine *TNF-α* fulfills an essential function in the anti-tumor response and is pivotal for eliciting a protective immune response. TNF-α overexpression is also an important cause of inflammatory pathology. During the cellular immune response, TNF-α protein is expressed rapidly, well before TNF-β (lymphotoxin A) and other cytokines. In human mononuclear cells from peripheral blood, levels of *TNF-α* mRNA reach their maximum within 3 h upon stimulation [12]. *TNF-α* pre-mRNA is spliced efficiently owing to an element of 104 nucleotides, the 2-aminopurine response element (2-APRE), located in the *TNF-α* 3′-UTR, well beyond the three introns [13] (Figure 1). This short RNA element renders the splicing of *TNF-α* mRNA susceptible to selective inhibition by the specific eIF2α kinase inhibitor, 2-aminopurine [12]. *TNF-α* pre-mRNA activates PKR even more effectively than long double-stranded RNA [13], the canonical activator of PKR [6]. The 2-APRE confers a regulatory advantage: it enhances the splicing of *TNF-α* mRNA by as much as 20-fold when the expression of PKR is increased [13]. The knockdown of PKR with antisense RNA abrogates *TNF-α* RNA splicing [10].

PKR forms a homodimer on the activating RNA; *trans*-autophosphorylation of the PKR dimer then leads to kinase activation and its release from the RNA [6,7] (Figure 1). The binding of PKR requires a minimum of 16–18 base pairs (bp) of double-stranded RNA and PKR activation of at least 33 bp, optimally around 80 bp [20,21]. This raises the question of how such a short RNA element could be so effective in activating PKR. The activator of PKR within *TNF-α* pre-mRNA is generated from helical domains that are each too short to be able to activate PKR directly; however, these domains can fold into a pseudoknot that is pivotal for PKR activation and promoting mRNA splicing, as shown by gain-of-function mutations [10]. The pseudoknot constrains the RNA into two double-helical stacks with parallel axes that are each long enough to engage a PKR monomer, thus allowing for the effective dimerization of the kinase, which results in PKR activation [10] (Figure 1). This compact pseudoknot structure, which is highly conserved within the phylogeny from turbot, a teleost fish, to humans, forms the molecular basis for the unusual ability of the 2-APRE element to activate PKR and thereby to render splicing of *TNF-α* mRNA highly efficient [10].

The activation of PKR by the 2-APRE achieves the highly efficient splicing of *TNF-α* mRNA through eIF2α phosphorylation [10] (Figure 1). The expression of eIF2αS51A, a non-phosphorylatable mutant of eIF2α that is mutated in Ser51, the sole phosphorylation site [9], yet not of the wild-type eIF2α, blocked the highly efficient splicing imparted by the presence of the 2-APRE [10]. By contrast, the expression of eIF2αS51D, a mutant containing a phosphomimetic D residue that inhibits translation [22], did not have a significant effect on splicing efficiency, thereby indicating the need for phosphorylated eIF2α. Unlike eIF2αS51D, eIF2αS51A strongly inhibits the phosphorylation of eIF2α by PKR that was activated by 2-APRE RNA in vitro [10]. On the other hand, the splicing of *TNF-β* pre-mRNA, which lacks an activator of PKR and is resistant to splicing inhibition by 2-AP [13], was rendered highly efficient when the overall level of phosphorylated eIF2α within the cell was elevated by the addition of salubrinal [10]. Salubrinal is a specific inhibitor of eIF2α dephosphorylation [16]. Globally raising phospho-eIF2α in the cell with salubrinal achieves high splicing efficiency even in the absence of an intragenic PKR activator [10]. Indeed, eIF2α phosphorylation enhances the splicing of *TNF-α* mRNA in primary human peripheral blood mononuclear cells that naturally express this cytokine [10]. Importantly, although the 2-APRE renders nuclear *TNF-α* mRNA splicing in cells highly efficient through the local activation of PKR, it does not induce the repression of translation in the cytoplasm [10,13] (Figure 1). Conceivably, the binding of cytoplasmic proteins to the TNF-α 3′-UTR after nuclear export acts, directly or indirectly, to mask the ability of the 2-APRE to activate PKR [13].

## 3. Positive Regulation of Splicing of *α-* and *β-Globin* mRNA by RNA Elements within the Pre-mRNA That Activate PKR, Inducing Nuclear eIF2α Phosphorylation

**Figure 2:**
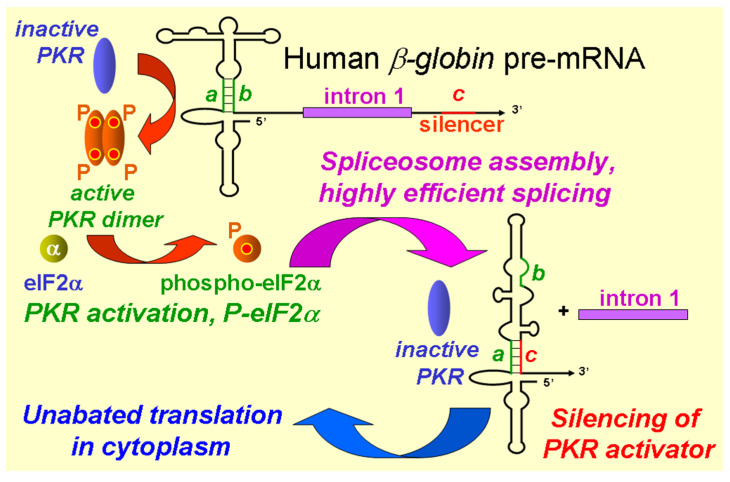
Control of splicing of *β-globin* pre-mRNA by an intragenic RNA element that activates PKR and silencing of the PKR activator upon excision of the first intron. Within the human *β-globin* RNA element that activates PKR, which consists of the 5′-terminal 124 nt of exon 1, the 5-bp helix *a-b* (green) plays a critical role in PKR activation. Once PKR has been activated, it must phosphorylate its eIF-2α substrate in order to promote the assembly of early spliceosomes and allow for the efficient splicing of *β-globin* mRNA. Once intron 1 is excised, sequence *c*, that is located close to the beginning of exon 2 (red) base-pairs with strand *a* and thereby displaces strand *b*, inducing a structural rearrangement within the RNA that results in silencing of the ability of the mature spliced *β-globin* mRNA to activate PKR. This structural rearrangement causes the activation of PKR to be transitory, serving only to enhance splicing while avoiding the inhibition of β-globin protein synthesis that is essential for survival [14,19].

To dissect the mechanism underlying the high splicing efficiency of *TNF-α* mRNA that is imparted through its intragenic RNA element that strongly activates PKR and mediated through the phosphorylation of eIF2α, *TNF-α* precursor RNA that was transcribed in vitro was used as a substrate for splicing in the nuclear extract of HeLa cells. This attempt failed because the *TNF-α* pre-mRNA substrate was promptly and completely degraded. However, these experiments led to the discovery that *β-globin* exon1-intron1-exon2 template, which was used as a standard positive control for in vitro splicing [23], also strictly requires the activation of PKR to undergo splicing [14]. That finding came as a surprise, given that the control of *globin* gene expression was investigated early in the development of molecular biology and has long served as a paradigm for the regulation of eukaryotic gene expression. Indeed, the activation of PKR that was induced by intragenic RNA activator elements within the human *α-globin*, *β-globin* and fetal *^A^γ-globin* pre-mRNA molecules is indispensable for their splicing [14] (Figure 2).

The splicing of *β-globin* mRNA in the intact cell is blocked by 2-aminopurine or by the co-expression of a dominant-negative mutant of PKR [14]. Similar to *IFN*-*γ* mRNA, *β-globin* pre-mRNA activates PKR in vitro [14]. The removal of *β-globin* intron 1, the first splicing event, is abrogated by antibodies against PKR as well as by the depletion of PKR; upon PKR depletion, splicing can be restored by the addition of recombinant human PKR [14]. The *β-globin* RNA element that activates PKR is located within the first exon (Figure 2); mutation of the small 5-bp helix *a-b* in the *β-globin* activator element strongly impairs activation of PKR as well as splicing of the mRNA. The efficient splicing of each of *α-*, *β-* and *^A^γ-globin* pre-mRNA species depends tightly on the activation of PKR and on the phosphorylation of eIF2α within the nucleus [14]. Splicing is inhibited by the expression of non-phosphorylatable mutant eIF2αS51A, as well as by antibodies, against phospho-eIF2α [14]. PKR is co-immunoprecipitated with splicing complexes and is required for spliceosome formation on *globin* pre-mRNA [14]. The activation of PKR kinase and phosphorylation of eIF2α promote an early step in the assembly of the *β-globin* spliceosome, formation of Complex A (Figure 2) [14].

During erythroid cell development, there is a massive translation of *globin* mRNA, reaching up to 95% of total protein within reticulocytes as compared to only <0.1% in proerythroblasts [24]. Considering that the *β-globin* activator of PKR is entirely contained within exon 1, in principle it could act, as for *IFN-γ* mRNA [9,11], to attenuate *β-globin* mRNA translation. Unabated translation after splicing is achieved via a novel mechanism. Removal of the first intron in *β-globin* pre-mRNA juxtaposes short sequence *c*, a 5-nucleotide sequence located close to the start of exon 2, to exon 1, inducing the displacement of strand *b* within exon 1 and thereby destroying essential helix *a-b* that constitutes the core of the PKR activator element and inducing a complete refolding of the RNA activator structure (Figure 2). This results in silencing of the activator of PKR as soon as the *β-globin* mRNA undergoes splicing (Figure 2) [14]. The splicing of *α-globin* pre-mRNA is regulated in a similar manner except that the PKR activator and the silencer are reversed in location between exons 2 and 1 [14]. The distinct locations of PKR activator and silencer elements within the *α-* and *β-globin* genes demonstrate evolutionary flexibility in terms of how the activation of PKR is controlled during splicing and once mRNA splicing has been completed. In fetal *^A^γ-globin* pre-mRNA, the sequences of activator of PKR and the silencer are located as within the homologous *β-globin* pre-mRNA [14]. This molecular mechanism ensures that the ability of *globin* pre-mRNA to activate PKR remains highly transient, serving only to render its splicing highly efficient, without impeding the synthesis of globin protein in the cytoplasm (Figure 2) [14].

These findings support the conclusion that the creation of intracellular stress, which induces eIF2α phosphorylation [1,2] that suffices to render splicing highly efficient [10], is beneficial for the effective splicing of *globin* mRNA, a key step towards the formation of hemoglobin.

## 4. Positive Control of Splicing of HIV *rev/tat* mRNA by RNA Elements within the Viral Genome That Activate PKR, a Cornerstone of the Antiviral Response

All mRNA species expressed by HIV contain at both 5′ and 3′ termini a 59-nucleotide stem-loop, the *trans*-activation response (TAR) RNA element, which was shown to be able to activate PKR in cell-free systems [25,26]. Until recently, it remained unknown whether TAR might activate PKR within the intact cell. However, once it became known that the activation of PKR fulfills an indispensable role in promoting the splicing of *TNF-α* and *globin* mRNA, this invited the hypothesis that the splicing of HIV mRNA potentially could be dependent on the activation of PKR by its precursor transcript, as well as on the phosphorylation of eIF2α. This turned out to be indeed the case. HIV has turned this major mechanism, the activation of PKR by the viral RNA, hitherto considered as a cornerstone of the cell’s antiviral response, used by the cell to protect itself from virus infection, into a tool to achieve splicing of the viral *rev/tat* mRNA, involving the excision of a large intron [18].

To investigate the possibility that the production of HIV mRNA might be mediated through the activation of PKR, the intact wild-type HIV-1 genome was expressed in human cells and the expression of distinct species of HIV mRNA was monitored following the addition of PKRi [18], a small molecule that inhibits the catalytic site in the PKR kinase [27]. The expression of all species of HIV mRNA, including not only singly spliced but also multiply spliced mRNAs, was i progressively inhibited by increasing doses of PKRi [18]. Moreover, the co-expression of a viral antagonist of PKR, the Vaccinia E3L protein [28], resulted in a broad inhibition of the production of mRNA species encoded by the viral genome [18]. The E3L protein competes with PKR in binding to the activating RNA, thereby creating an E3L-PKR-RNA complex wherein the N-terminal half of the E3L molecule binds directly into the protein kinase catalytic domain of PKR [28]. The inhibition that was observed independently with PKRi, a small-molecule inhibitor of PKR activation, and with a viral antagonist of PKR, Vaccinia E3L, supported the concept that PKR activation plays a critical role in the control of HIV mRNA expression [18]. A mutant HIV-1 genome construct lacking TAR remained fully sensitive to PKRi, suggesting that the various pre-mRNA species encoded by the HIV genome might harbor, in addition to the TAR element, another potential activator of PKR [18].

**Figure 3:**
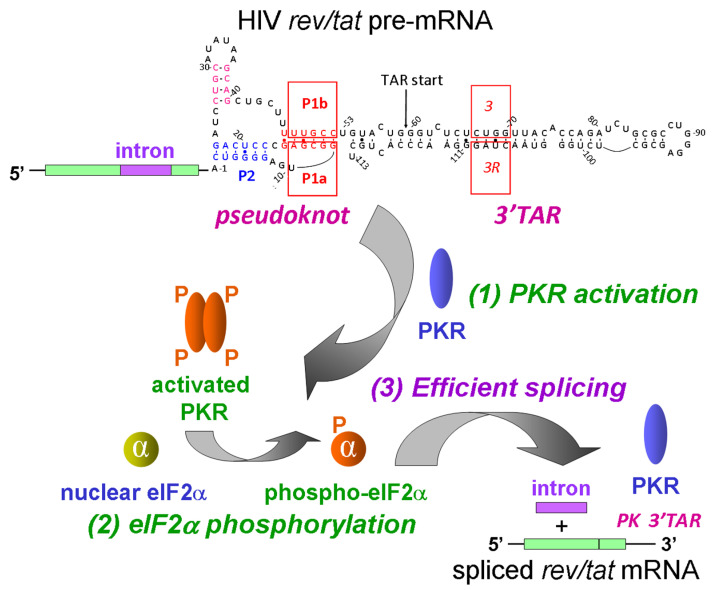
Splicing of HIV *rev/tat* mRNA is dependent on the activation of PKR by an intragenic pseudoknot located just upstream of 3′TAR and by the 3′TAR stem-loop, inducing eIF2α phosphorylation [18]. Local activation of PKR within the cell, predominantly by the pseudoknot element (PK) yet also by 3′TAR (*1*), induces eIF2α phosphorylation (*2*) that triggers excision of the large *rev/tat* intron (*3*). Mutations (boxed) within pseudoknot stem P1 or within the stem of 3′TAR each cause a severe impairment in splicing of *rev/tat* mRNA [18].

It is difficult to interpret effects on splicing by analysis of the full-length HIV genome, because a decrease in mRNA splicing will also lead to a decrease in the expression of the Tat and Rev proteins that are both encoded by mRNAs that undergo multiple splicing [29]. Tat not only activates viral transcription but also regulates splicing of the viral RNA species [30,31]. Rev, on the other hand, stimulates the nuclear export of both unspliced and singly spliced RNAs to cause reduced splicing [32,33]. These properties will mask a specific effect on splicing. The inhibition of splicing may lead to the degradation of unspliced RNA precursor transcripts that cannot proceed to export from the nucleus. Hence, to resolve the molecular mechanism that underlies the need for PKR activation in splicing of HIV RNA, an expression vector was constructed that harbors 40% of the HIV genome, including its entire 3′ domain sequence [18]. The use of this vector enabled the analysis of the removal of the large *rev/tat* intron in the absence of confounding effects of the viral proteins, Tat and Rev [18]. The co-expression of Vaccinia E3L [28] and Ebola VP35 [34], viral proteins that are used to prevent the activation of PKR and thereby to evade the antiviral response that is induced by interferon, caused a severe inhibition of intron excision from HIV *rev/tat* pre-mRNA [18]. *Rev/tat* mRNA splicing was also inhibited severely by the co-expression of a *trans*-dominant negative mutant of PKR that blocks the phosphorylation of the kinase that is essential for its activation [18]. By contrast, splicing was stimulated significantly when PKR was overexpressed in the cell [18]. Hence, the activation of PKR is needed for *rev/tat* mRNA splicing (Figure 3, step 1). The efficient splicing of *rev/tat* mRNA was abrogated upon the expression of non-phosphorylatable mutant eIF2αS51A, yet was left fully intact when wild-type eIF2α was expressed [18]. By contrast, the expression of the phosphomimetic mutant eIF2αS51D had no inhibitory effect on splicing of *rev/tat* mRNA [18], demonstrating the need for authentic phosphorylated eIF2α (Figure 3, steps 2 and 3).

Bioinformatic analysis followed by mutational probing revealed the existence of an RNA pseudoknot that is located upstream of the 3′-terminal TAR stem-loop; by activating PKR, this compact RNA pseudoknot enables splicing of *rev/tat* mRNA [18] (Figure 3, top). This small pseudoknot escaped detection by chemical probing analysis of the full-length genomic HIV-1 RNA [35]. However, chemical probing of the RNA sequence just upstream of TAR is compatible with two pseudoknot stems with moderate stability, as would be expected if the compact pseudoknot undergoes dynamic refolding [18]. Mutations made in pseudoknot stem P1 or within the stem of 3′TAR each impair splicing [18] (Figure 3).

Manmade TAR mutation 3 and compensatory mutation 3R each impair the activation of PKR in vitro, whereas double-mutation 3R3 restores PKR activation [25]. Indeed, in intact cells, the splicing efficiency of *rev/tat* pre-mRNA was reduced by each of the mutations 3 and 3R, although less severely than by manmade mutation P1b, and was restored in part by the double mutation 3R3 that restores base pairing, albeit in the opposite orientation [18].

The RNA pseudoknot and the 3′-terminal TAR structure play a collaborative role in mediating the PKR-dependent splicing of *rev/tat* mRNA, wherein the pseudoknot is the dominant element, as judged from the more severe impact of pseudoknot mutations on splicing [18] (Figure 3, steps 1 and 3). Thus, unlike the case for *TNF-α* and *globin* pre-mRNA, the HIV-1 RNA activator of PKR that drives splicing of *rev/tat* pre-mRNA is composite in nature. Conceivably, to ensure efficient splicing, HIV evolved to harbor dual RNA activators of PKR.

The formation of the RNA pseudoknot is conserved amongst diverse isolates of HIV that belong to distinct subtypes of HIV group M (which comprises well over 90% of all human HIV/AIDS cases) as well as in nonhuman primate SIVcpz isolates (where the RNA can fold into a comparable pseudoknot), supporting the concept that it fulfills an essential function in the regulation of mRNA splicing [18].

As noted above, splicing effects that are based on an analysis of the expression of the complete HIV genome are hard to interpret. It is worth mentioning, within this context, that a number of breakthrough discoveries regarding the regulation of HIV-1 gene expression were made possible through an experimental approach in which a vector that expresses only a part of the full HIV-1 genome in cell lines was used to dissect the molecular mechanism that underlies the control of viral gene expression [36,37,38,39]. This consideration strengthens the concept that the PKR-dependent splicing mechanism schematized in Figure 3 is directly relevant to the control of gene expression during replication of the intact HIV-1 virus [18].

## 5. Discussion and Conclusions

The concept that short, noncoding RNA elements within genes can attenuate mRNA translation or enhance pre-mRNA splicing in *cis*, respectively, by activating PKR and inducing the phosphorylation of eIF2α emerged from studies of the inflammatory cytokine genes that encode *IFN-γ* [9,11] and *TNF-α* [10,13]. Future work will show how frequently the activation of PKR by structures within pre-mRNA is used to facilitate the splicing of mRNA. The data reviewed above support the growing impact of PKR-dependent splicing. The transient activation of PKR by short intragenic sequences within unspliced pre-mRNA, resulting in the phosphorylation of eIF2α, not only enables the highly efficient splicing of cellular mRNA as exemplified by the *TNFα* gene and the *globin* genes that are essential for survival (Figure 1 and Figure 2), but can even serve to enable the splicing of a viral mRNA, as exemplified for the first time by the *rev/tat* mRNA of HIV (Figure 3). As such, the activation of PKR by viral RNA, considered a key element of the antiviral response of the host cell, can positively regulate the splicing of viral mRNA. Future research may show whether the example of HIV represents an isolated case or is used more widely among viruses.

To examine how extensively the RNA splicing of cellular genes is controlled via the activation of PKR and the phosphorylation of eIF2α, one may consider performing transcriptome-wide RNAseq experiments upon the depletion of PKR, the use of eIF2αS51A expression or PKRi, the drug that targets this kinase. This approach should be the fastest way to detect cellular RNAs whose splicing is under control of this pathway, while a combination of this with an array of intron-containing viruses (negative single-stranded and double-stranded RNA or even DNA) should reveal which viruses, other than HIV, co-opt this mechanism.

It is notable that the activators of PKR within *IFN-γ* mRNA [9,11], *TNF-α* pre-mRNA [10] as well as in HIV *rev/tat* pre-mRNA [18] consist of compact pseudoknotted RNA structures. Accordingly, a broader examination of the occurrence of the pseudoknot fold within RNA encoded by cellular genes, as well as by viral genomes, may well provide useful leads for future research focused on finding novel instances of PKR-dependent mRNA splicing. However, it also is clear that a pseudoknot is not necessarily essential for PKR activation, as witnessed by the ability of the short 59-nt HIV TAR element to activate PKR by means of an extended RNA stem (Figure 3). Likewise, it is common knowledge that the double-stranded RNA generated within the cell upon replication of viral RNA during infection acts as a major activator of PKR, and that this underlies the IFN-induced cellular antiviral response.

The need for activation of PKR and the phosphorylation of eIF2α imparted by the intragenic RNA elements reviewed above links the splicing of mRNA to the integrated cellular stress response, which is characterized by the phosphorylation of eIF2α [1,2]. The existence of RNA activators of PKR within diverse gene sequences that are essential for splicing of the encoded mRNA [10,13,14,18] shows that stress can upregulate gene expression at the mRNA-splicing stage via the activation of PKR.

The production of hemoglobin depends on the massive expression of *globin* mRNAs in erythroid cells [24] that demands the highly efficient splicing of *globin* precursor transcripts. How the assembly of early spliceosomes is promoted by the phosphorylation of eIF2α, as documented for *β-globin* pre-mRNA [14], remains a subject for future research.

## Data Availability

Not applicable.

## References

[1] Harding H.P., Zhang Y., Zeng H., Novoa I., Lu P.D., Calfon M., Sadri N., Yun C., Popko B., Paules R. (2003). An integrated stress response regulates amino acid metabolism and resistance to oxidative stress. Mol. Cell.

[2] Muaddi H., Majumder M., Peidis P., Papadakis A.I., Holcik M., Scheuner D., Kaufman R.J., Hatzoglou M., Koromilas A.E. (2010). Phosphorylation of eIF2α at serine 51 is an important determinant of cell survival and adaptation to glucose deficiency. Mol. Biol. Cell.

[3] Sonenberg N., Hinnebusch A.G. (2009). Regulation of translation initiation in eukaryotes: Mechanisms and biological targets. Cell.

[4] Stark G.R., Kerr I.M., Williams B.R., Silverman R.H., Schreiber R.D. (1998). How cells respond to interferons. Annu. Rev. Biochem..

[5] Kaufman R.J. (1999). Double-stranded RNA-activated protein kinase mediates virus-induced apoptosis: A new role for an old actor. Proc. Natl. Acad. Sci. USA.

[6] Zhang F., Romano P.R., Nagamura-Inoue T., Tian B., Dever T.E., Mathews M.B., Ozato K., Hinnebusch A.G. (2001). Binding of double-stranded RNA to protein kinase PKR is required for dimerization and promotes critical autophosphorylation events in the activation loop. J. Biol. Chem..

[7] Dey M., Cao C., Dar A.C., Tamura T., Ozato K., Sicheri F., Dever T.E. (2005). Mechanistic link between PKR dimerization; autophosphorylation; and eIF2α substrate recognition. Cell.

[8] Launer-Felty K., Wong C.J., Cole J.L. (2015). Structural analysis of adenovirus VAI RNA defines the mechanism of inhibition of PKR. Biophys. J..

[9] Ben-Asouli Y., Banai Y., Pel-Or Y., Shir A., Kaempfer R. (2002). Human interferon-γ mRNA autoregulates its translation through a pseudoknot that activates the interferon-inducible protein kinase PKR. Cell.

[10] Namer L.S., Osman F., Banai Y., Masquida B., Jung R., Kaempfer R. (2017). An ancient pseudoknot in TNF-α pre-mRNA activates PKR; inducing eIF2α phosphorylation that potently enhances splicing. Cell Rep..

[11] Cohen-Chalamish S., Hasson A., Weinberg D., Namer L.S., Banai Y., Osman F., Kaempfer R. (2009). Dynamic refolding of IFN-γ mRNA enables it to function as PKR activator and translation template. Nat. Chem. Biol..

[12] Jarrous N., Osman F., Kaempfer R. (1996). 2-Aminopurine selectively inhibits splicing of tumor necrosis factor α mRNA. Mol. Cell. Biol..

[13] Osman F., Jarrous N., Ben-Asouli Y., Kaempfer R. (1999). A cis-acting element in the 3′-untranslated region of human TNF-α mRNA renders splicing dependent on the activation of protein kinase PKR. Genes Dev..

[14] Ilan L., Osman F., Namer L.S., Eliahu E., Cohen-Chalamish S., Ben-Asouli Y., Banai Y., Kaempfer R. (2017). PKR activation and eIF2α phosphorylation mediate human globin mRNA splicing at spliceosome assembly. Cell Res..

[15] Tan S.L., Tareen S.U., Melville M.W., Blakely C.M., Katze M.G. (2002). The direct binding of the catalytic subunit of protein phosphatase 1 to the PKR protein kinase is necessary but not sufficient for inactivation and disruption of enzyme dimer formation. J. Biol. Chem..

[16] Boyce M., Bryant K.F., Jousse C., Long K., Harding H.P., Scheuner D., Kaufman R.J., Ma D., Coen D.M., Ron D. (2005). A selective inhibitor of eIF2α dephosphorylation protects cells from ER stress. Science.

[17] Tsaytler P., Harding H.P., Ron D., Bertolotti A. (2011). Selective inhibition of a regulatory subunit of protein phosphatase 1 restores proteostasis. Science.

[18] Namer L.S., Harwig A., Heynen S.P., Das A.T., Berkhout B., Kaempfer R. (2023). HIV co-opts a cellular antiviral mechanism, activation of stress kinase PKR by its RNA, to enable splicing of rev/tat mRNA. Cell Biosci..

[19] Kaempfer R., Ilan L., Cohen-Chalamish S., Turgeman O., Namer L.S., Osman F. (2019). Control of mRNA splicing by intragenic RNA activators of stress signaling: Potential implications for human disease. Front. Genet..

[20] Bevilacqua P.C., Cech T.R. (1996). Minor-groove recognition of double-stranded RNA by the double-stranded RNA-binding domain from the RNA-activated protein kinase PKR. Biochemistry.

[21] Manche L., Green S.R., Schmedt C., Mathews M.B. (1992). Interactions between double-stranded RNA regulators and the protein kinase DAI. Mol. Cell. Biol..

[22] Srivastava S.P., Kumar K.U., Kaufman R.J. (1998). Phosphorylation of eukaryotic translation initiation factor 2 mediates apoptosis in response to activation of the double-stranded RNA-dependent protein kinase. J. Biol. Chem..

[23] Krainer A.R., Maniatis T., Ruskin B., Green M.R. (1984). Normal and mutant human β-globin pre-mRNAs are faithfully and efficiently spliced in vitro. Cell.

[24] Nienhuis A.W., Benz E.J. (1977). Regulation of hemoglobin synthesis during the development of the red cell. N. Engl. J. Med..

[25] Edery I., Petryshyn R., Sonenberg N. (1989). Activation of double-stranded RNA-dependent kinase (dsl) by the TAR region of HIV-1 mRNA: A novel translational control mechanism. Cell.

[26] Ben-Asouli Y., Banai Y., Hauser H., Kaempfer R. (2000). Recognition of 5’-terminal TAR structure in human immunodeficiency virus-1 mRNA by eukaryotic translation initiation factor 2. Nucleic Acids Res..

[27] Jammi N.V., Whitby L.R., Beal P.A. (2003). Small molecule inhibitors of the RNA-dependent protein kinase. Biochem. Biophys. Res. Commun..

[28] Romano P.R., Zhang F., Tan S.L., Garcia-Barrio M.T., Katze M.G., Dever T.E., Hinnebusch A.G. (1998). Inhibition of the double-stranded RNA-dependent protein kinase PKR by Vaccinia virus E3: Role of complex formation and the E3 N-terminal domain. Mol. Cell. Biol..

[29] Karn J., Stoltzfus C.M. (2012). Transcriptional and posttranscriptional regulation of HIV-1 gene expression. Cold Spring Harb. Perspect. Med..

[30] Jablonski J.A., Amelio A.L., Giacca M., Caputi M. (2010). The transcriptional transactivator Tat selectively regulates viral splicing. Nucleic Acids Res..

[31] Mueller N., Pasternak A.O., Klaver B., Cornelissen M., Berkhout B., Das A.T. (2018). The HIV-1 Tat protein enhances splicing at the major splice donor site. J. Virol..

[32] Fischer U., Huber J., Boelens W.C., Mattaj I.W., Lührmann R. (1995). The HIV-1 Rev activation domain is a nuclear export signal that accesses an export pathway used by specific cellular RNAs. Cell.

[33] Yedavalli V.S., Neuveut C., Chi Y.H., Kleiman L., Jeang K.T. (2004). Requirement of DDX3 DEAD box RNA helicase for HIV-1 Rev-RRE export function. Cell.

[34] Schümann M., Gantke T., Mühlberger E. (2009). Ebola virus VP35 antagonizes PKR activity through its C-terminal interferon inhibitory domain. J. Virol..

[35] Wats J.M., Dang K.K., Gorelick R.J., Leonard C.W., Bess J.W., Swanstrom R., Burch C.L., Weeks K.M. (2009). Architecture and secondary structure of an entire HIV-1 RNA genome. Nature.

[36] Kao S.Y., Calman A.F., Luciw P.A., Peterlin B.M. (1987). Anti-termination of transcription within the long terminal repeat of HIV-1 by tat gene product. Nature.

[37] Berkhout B., Gatignol A., Rabson A.B., Jeang K.T. (1990). TAR-independent activation of the HIV-1 LTR: Evidence that Tat requires specific regions of the promoter. Cell.

[38] Selby M.J., Peterlin B.M. (1990). Trans-activation by HIV-1 Tat via a heterologous RNA binding protein. Cell.

[39] Gatignol A., Buckler-White A., Berkhout B., Jeang K.T. (1991). Characterization of a human TAR RNA-binding protein that activates the HIV-1 LTR. Science.

